# Exosome-Mediated Delivery of the Neuroprotective Peptide PACAP38 Promotes Retinal Ganglion Cell Survival and Axon Regeneration in Rats With Traumatic Optic Neuropathy

**DOI:** 10.3389/fcell.2021.659783

**Published:** 2021-04-06

**Authors:** Tian Wang, Yiming Li, Miao Guo, Xue Dong, Mengyu Liao, Mei Du, Xiaohong Wang, Haifang Yin, Hua Yan

**Affiliations:** ^1^Department of Ophthalmology, Tianjin Medical University General Hospital, Tianjin, China; ^2^Laboratory of Molecular Ophthalmology, Tianjin Medical University, Tianjin, China; ^3^Tianjin Key Laboratory of Inflammation Biology, Department of Pharmacology, School of Basic Medical Sciences, Tianjin Medical University, Tianjin, China; ^4^Tianjin Key Laboratory of Cellular Homeostasis and Human Diseases, Department of Cell Biology, Tianjin Medical University, Tianjin, China

**Keywords:** traumatic optic neuropathy, exosomes, PACAP38, axon regeneration, retina ganglion cell survival

## Abstract

Traumatic optic neuropathy (TON) refers to optic nerve damage caused by trauma, leading to partial or complete loss of vision. The primary treatment options, such as hormonal therapy and surgery, have limited efficacy. Pituitary adenylate cyclase-activating polypeptide 38 (PACAP38), a functional endogenous neuroprotective peptide, has emerged as a promising therapeutic agent. In this study, we used rat retinal ganglion cell (RGC) exosomes as nanosized vesicles for the delivery of PACAP38 loaded via the exosomal anchor peptide CP05 (EXO_*PACAP38*_). EXO_*PACAP38*_ showed greater uptake efficiency *in vitro* and *in vivo* than PACAP38. The results showed that EXO_*PACAP38*_ significantly enhanced the RGC survival rate and retinal nerve fiber layer thickness in a rat TON model. Moreover, EXO_*PACAP38*_ significantly promoted axon regeneration and optic nerve function after injury. These findings indicate that EXO_*PACAP38*_ can be used as a treatment option and may have therapeutic implications for patients with TON.

## Introduction

Traumatic optic neuropathy (TON) refers to optic nerve damage secondary to trauma, and leads to partial and complete loss of vision. TON is one of the most severe eye traumas, accounting for 0.5–5% of all craniocerebral traumas (Pirouzmand, 2012). Intraductal optic nerve injury is the most common cause of TON owing to the anatomical structure and physiological characteristics of the region (Ganguly and Barik, 2015). TON can result in axonal damage, leading to the gradual irreversible loss of retinal ganglion cells (RGCs) and, consequently, to permanent visual deficiency. Currently, no effective treatment is available for TON (Chaon and Lee, 2015; Singman et al., 2016; Yan et al., 2016). The clinical treatment options for TON include observation (conservative management), high-dose corticosteroid treatment, or surgery (optic canal decompression), which are based on studies on small patient cohorts (Goldberg and Steinsapir, 1996; Yu-Wai-Man and Griffiths, 2013; Yan et al., 2016; Yu et al., 2016; Kashkouli et al., 2017). In comparative nonrandomized studies on the treatment outcomes in patients with TON, the results showed no clear effect of either hormonal therapy or surgical treatment (Levin et al., 1999; Carta et al., 2003). Therefore, more effective therapies to restore the vision of patients with TON are urgently needed (Bastakis et al., 2019).

Pituitary adenylate cyclase-activating polypeptide (PACAP) is an endogenous neuropeptide originally identified in the hypothalamus of sheep, and was named after its ability to activate adenylate cyclase in rat pituitary cells (Miyata et al., 1989). PACAP, as a neurotransmitter, neuromodulator, or neurotrophic factor, plays an important role in neuronal development and regeneration, and may possess potent neuroprotective effects under pathophysiological conditions (White et al., 2010; Sherwood et al., 2016). It regulates various physiological processes through two different types of receptors: PACAP receptor type 1 (PAC1R) and vasoactive intestinal peptide/PACAP receptor (Rampelbergh et al., 1997). Previous studies have shown that PACAP and its receptors are widely distributed in brain tissues, and that PACAP plays a neuroprotective role in neurodegenerative diseases such as stroke (Matsumoto et al., 2016), traumatic brain injury (Toth et al., 2020), Alzheimer’s disease (Han et al., 2014), and Parkinson’s disease (Wang et al., 2008). Previous observations have revealed that PACAP is expressed in the ganglion cell layer (GCL) and in the body of amacrine and horizontal cells. Moreover, it was also found to be expressed in the nerve fiber layer (NFL) and inner plexiform layer (INL) of the rat retina (Atlasz et al., 2010). Endogenous PACAP has been reported to be involved in the development of neurodegeneration in optic nerve crush (ONC) injury, which closely mimics the axonal degeneration of RGCs and subsequent loss of RGCs in TON (Tang et al., 2011). PACAP and its receptor PAC1R are primarily expressed in the GCL, and their expression has been shown to undergo spatiotemporal changes in ONC rats. Intravitreal injections of PACAP38 have been shown to decrease the apoptosis of RGCs at 7 days after injury in ONC rats (Ye et al., 2019). However, PACAP38 has the drawbacks of insufficient uptake and need for repeated injections. Therefore, an efficient delivery system may be able to overcome this limitation.

Exosomes are membranous nanovesicles with a diameter of 50–150 nm that are secreted by various types of cells after the fusion of multiple vesicular bodies with the plasma membrane (Hessvik and Llorente, 2017). Exosomes are known to be intercellular messengers whose cargo, containing proteins, lipids, and nucleic acids, could be delivered into recipient cells (Colombo et al., 2014). In the retina, exosomes present multiple advantages over existing synthetic systems for the treatment of posterior ocular diseases through intravitreal injection. First, exosomes show low immunogenicity, which can avoid the vitreous opacity or secondary retinal damage caused by the hyperplastic membrane formed by proliferating cells (Kuriyan et al., 2017). Second, the phospholipid bilayer of exosomes may fuse with the target cell plasma membrane and bypass the endosomal-lysosomal pathway utilized by other synthetic nanoparticles, which can activate the inflammasomes (Hornung et al., 2008; Tatischeff and Alfsen, 2011). Moreover, the size of exosomes might be beneficial for the treatment of TON, as studies have shown that only small particles (50–200 nm) could reach the retina after intravitreal injection, whereas micron-sized particles usually remain in the trabecular meshwork and vitreous cavity (Barza et al., 1987; Sakurai et al., 2001).

In this study, we aimed to investigate the feasibility of using exosomes derived from rat RGCs (EXOs) as an ideal system for the delivery of PACAP38 to the retina, to improve the barrier penetration capacity and stability of PACAP38. CP05 has been demonstrated to function as an exosomal anchor peptide by binding to the exosomal surface protein CD63, which is a tetraspanin present in large amounts on the exosome surface and has been used as an exosomal marker (Gao et al., 2018). By using this system, we have successfully loaded an anti-angiogenic peptide for ocular delivery to treat proliferative retinopathy (Dong et al., 2021). In this study, we aimed to load PACAP38 onto exosomes via CP05, and to evaluate whether this systemic EXO_*PACAP38*_ can mediate effective neuroprotection and axon regeneration in TON rats.

## Materials and Methods

### Exosome Isolation and Identification

The supernatants of RGC culture medium were collected in polypropylene centrifuge tubes and centrifuged at 300 g at 4°C for 10 min, aimed at removing the free cells. Then supernatants were transferred to fresh polypropylene tubes. It was centrifuged at 2,000 g at 4°C for 10 min to remove the cell debris and again at 10,000 g at 4°C for 30 min, aimed at further removing the cell particles. Next, the supernatants were filtered through a 0.22 mm filter to remove the particles larger than 200 nm and dead cells. Finally, it was ultracentrifuged at 100,000 g at 4°C for 70 min to collect the exosomes. The supernatants were discarded, and the pellets were resuspended to an appropriate concentration with 0.9% sodium chloride solution that has been centrifuged and stored at -80°C for further experiments.

Exosomal size distribution was detected and analyzed by by Nanosight NS300 (Malvern, UK) strictly following the manufacturer’s instructions. The morphology of particles was examined using a transmission electron microscopy (TEM, HT7700; Hitachi, Tokyo, Japan). Biomarkers for exosomes including CD63(Cat#sc-5275, Santa Cruz, United States), CD81 (Cat#sc-166029, Santa Cruz, United States), CD9 (Cat#ab92726, abcam), Alix (Cat#2171, Cell Signaling Technology), and Cytochrome C (Cat#11940, Cell Signaling Technology, United States) were detected with Western blot analysis.

### Cellular Uptake *in vitro*

To test the cellular uptake of EXO with PACAP38 and EXO_*PACAP38*_, DiR-labeled exosomes (1 μg) were incubated with FITC-labeled CP05-PACAP38 (20 μM) or FITC-labeled PACAP38 (20 μM) at 4°C for 6 h. Isolated exosomes from rat RGCs were labeled with DiR (Invitrogen, United States). Subsequently, peptides and exosomes mixture or peptide-exosome complexes were incubated with RGCs for 24 or 48 h. Cells were washed with cold phosphate-buffered saline (PBS) and fixed with 4% PFA for 30 min at RT and stained with DAPI. Images were obtained by confocal microscope (LSM800, Cari Zeiss, Germany). To compare peptide delivery efficiency, cells of each group were harvested and then analyzed with fluorescence-activated cell sorting (FACS, Verse, BD, United States).

### Animals

8-week-old male Sprague-Dawley (SD) rats (weighing 180–200 g) were purchased from the Chinese Academy of Military Science (Beijing, China). All experimental procedures were approved by the Tianjin Medical University Animal Care and Use Committee.

### Retinal Uptake *in vivo*

To test the distribution of PACAP38 and EXO_*PACAP38*_ in the retina, PACAP38 and EXO_*PACAP38*_ was administered intravitreally into SD rats. Rhodamine-labeled CP05-PACAP38 (20 μM) were incubated with EXO at 4°C for 6 h in saline solution (0.9% NaCl). Rhodamine-labeled PACAP38 (20 μM) or rhodamine-labeled EXO_*PACAP38*_ (20 μM) was injected intravitreally. After 2 or 6 h, rats were sacrificed, and the eyeballs were harvested and fixed in 4% PFA for 1 h at 4°C. Afterward, eyeballs were enucleated in PBS and transferred to 30% sucrose/PBS at 4°C overnight and embedded in optimal cutting temperature compound (Sakura, Japan) and frozen. Serial 20 μm-thick sections were cut using a cryostat (CM1950, Leica, Germany). Cryosections were washed, then permeabilized and blocked in 5%BSA, 0.5% PBST (PBS with Triton X-100). Sections were incubated in rabbit anti-RBPMS antibody (1:200, Cat#ab152101, abcam) dissolved in 1% goat serum in 0.1% PBST overnight, then washed with PBS and incubated with anti-rabbit Alexa Fluor 594 (1:400; Cat #111-585-003, Jackson ImmunoResearch) and DAPI. Images were collected on a confocal microscope (LSM900, Carl Zeiss, Germany).

### Optic Nerve Crush and Intraocular Injection

The rats were anesthetized with 5% isoflurane/1.5 liter per minute O_2_ and maintained 3% throughout the procedure. The eye injection and ONC were performed as previously described (Mead and Tomarev, 2017). Briefly, the optic nerve was exposed intraorbitally by blunt dissection and crushed with reverse microscopic self-closing forceps (Dumont #N7, Roboz, Cat #RS-5027) for 10 s at a point∼1.5 mm posterior to the optic disk. Extreme care was taken not to damage the ocular blood vessels. Control rats underwent the same procedures except for the ONC. For intravitreal injections, a Hamilton syringe needle (33G) was inserted into the peripheral retina, taking care to avoid damaging the lens. 4 μL of EXO, PACAP38, or EXO_*PACAP38*_ (20 μM) were intravitreally injected after crush and 7 days after injury. Animals were sacrificed 7 days or 14 days after injury, and their retinae and optic nerves were harvested.

### Quantification of RGC Survival

Eyeballs were dissected and fixed with PFA (4%) for 30 mins at room temperature. Wholemount retina eyecups were permeabilized with 1% PBST and blocked in 5% goat serum in 0.5% PBST. Next, the wholemount was incubated for 2 days on a shaker at 4°C in rabbit anti-RBPMS antibody (1:200, Cat#ab152101, abcam) dissolved in 1% goat serum in 0.1% PBST. After being washed five times by 0.1% PBST, the retinal wholemount was incubated with anti-rabbit Alexa Fluor 594 (1:400; Cat #111-585-003, Jackson ImmunoResearch) 2 h at room temperature, protect from light. Finally, after being washed six times with 0.1% Triton-X100 in PBS at room temperature, protect from light, and then flat mounted. Images of flat-mounted retinae were taken using a × 10 objective with tile scans with Z-stacks on a Zeiss LSM800 confocal microscope. Fiji software was used to count RBPMS^+^ cells per retina from 12 fluorescence images taken at specific areas with one square millimeter, including four at 0.5 mm, four at 1.5 mm, and four at 2.5 mm from the optic nerve head, and overall RGCs survival was estimated.

### Measurement of the Retinal Nerve Fiber Layer (RNFL) Thickness

Animals were administered intraperitoneal anesthesia with 10% chloral hydrate based on their body weight (300 mg/kg). Next, rats were given atropine eye drops to dilated pupils for 10 min, then surface anesthesia with Promethazine Hydrochloride Caine eye drops was applied to the target eye for 5 min. Finally, transparent eye gel is covered on the cornea of both eyes to keep the cornea moist. Optical coherence tomography (OCT) imaging and analyzing were performed on rats under above anesthesia pre-injury, 7 and 14 days after injury, before sacrifice and tissue collection. The images of the rat retina around the optic nerve head were captured and measured by a Phoenix Micron IV Retinal Imaging Microscope (United States). Its in-built software was used to segment the RNFL and quantify the thickness. Segmentation could be manually adjusted when necessary to prevent the inclusion of blood vessels populated by the RNFL.

### Quantification of Axon Regeneration

Alexa Fluor^®^ 488-conjugated Cholera Toxin B (Cat#C34778, Thermo Fisher Scientific, Waltham, MA) was injected into the rat vitreous at 12 days after ONC surgery for anterograde labeling of the regenerated axons. Optic nerves were dissected and fixed with 4% paraformaldehyde in PBS overnight. The optic nerve was infiltrated in FocusClear^TM^ (CelExplorer, Hsinchu, Taiwan) for 6 h to make the tissue completely transparent. The whole nerve is then transferred into a small chamber built on the load glass slide aimed at providing enough space for the tissue and protecting it from squashed. Finally, the optic nerve in the chamber was coated in MountClear^TM^ mounting media (CelExplorer, Hsinchu, Taiwan), and the cover glass slide was coverslipped. Cleared optic nerve was imaged and analyzed with a confocal microscope by scanning each optical slice of different levels. A total of 7–15 optical slices were scanned for each optic nerve, and stacked optical images were captured at 10 μm intervals. The number of CTB-labeled regenerated axons at specific distance from the injury site were measured in at least three optical sections from the individual cases and analyzed with the formula as described previously (Leon et al., 2000).

$$\sum \text{ad}=\pi \,{\text{r}}^{2}*\left[\frac{\mathrm{Average}\,\mathrm{number}\,\mathrm{of}\,\mathrm{axons}}{\mathrm{mm}\,\mathrm{width}}\right]/\mathrm{Section}\,\mathrm{thickness}$$

The virtual thickness of per optical slice imaged with confocal microscope was analyzed using the formula as described previously (Leon et al., 2000).

$$\text{dz}≅\frac{0.64*\lambda \,\text{exc}}{n-\sqrt{{\text{n}}^{2}}-N\,{\text{A}}^{2}}$$

We calculated that the thickness of an optical section was 4.6 μm, in which the excitation wavelength was 488 nm, the refractive index (n) of the cover glass slide was 1.517. Our numerical aperture (Na) was 0.45. As the optical section’s virtual thickness was 4.6 μm, which is less than 10 μm intervals between the optical sections, single axons were not analyzed multiple times. For quantifying the number of axons at 0.5 mm from injury site, as there are very few axons observed in some cases, the optic nerve axons’ counts were used as the evaluation index of regeneration.

### Flash Visual Evoked Potentials (F-VEP) Recording

An RETI-port/scan 21 vision electrophysiological diagnostic apparatus (Roland Consult, German) was used, following the International Society for the Clinical Electrophysiology of Vision (ISCEV) standard electrophysiological studies. After 15 min of dark adaptation, Animals were administered intraperitoneal anesthesia with 10% chloral hydrate based on their body weight (300 mg/kg). F-VEP were recorded using silver needle electrodes, which were implanted under the skin in the middle of two ears. A reference electrode was implanted into the cheek of the recorded side, and the ground electrode was implanted into the tail of the rat. White flash stimuli were delivered at a frequency of 1.4 Hz, 250–500 ms for analysis, and superposition was conducted 100 times. Stable waveforms were recorded three times in each eye, and the contralateral eye was shaded with an eyeshade. The parameters observed were F-VEP latency (P2 wave response time, ms), N2-P2 amplitude (from N2 wave to P2 trough wave peak, mV). All parameter values were measured automatically by computer output, and the average of the three measurements was calculated.

### Statistics

All data are expressed as means ± SEM. Statistical differences between control and experimental groups were analyzed by Prism. Both parametric (used for samples with a normal distribution) and nonparametric (performed for samples on a non-normal distribution) analyses were evaluated. Statistical comparison between at least three groups was analyzed with one-way analysis of variance (ANOVA). A value of *P* < 0.05 was considered significant.

## Results

### Exosomes Mediate Efficient Uptake of PACAP38 *in vitro* and *in vivo*

**FIGURE 1:**
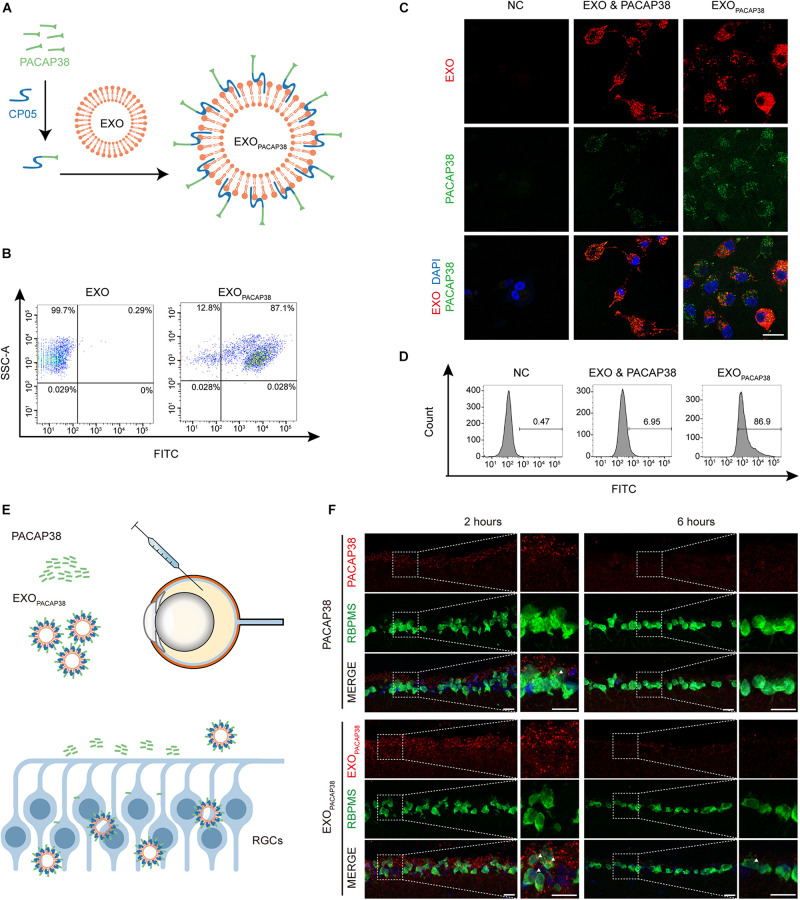
Efficient uptake of EXO_*PACAP38*_ *in vitro* and *in vivo*. **(A)** Scheme of preparing the PACAP38 delivery system, EXO_*PACAP38*_. **(B)** Results of FACS assessing the binding efficiency of PACAP38 on EXOs via the exosome anchor peptide CP05. CP05 and PACAP38 form the chimera CP05-PACAP38 through a chemical method *in vitro*, and CP05-PACAP38 was labeled with FITC. **(C)** Representative fluorescence microscopic images showing the uptake of PACAP38 in RGCs. PACAP38 and CP05-PACAP38 were labeled with FITC, and the EXOs were labeled with DiR (scale bar = 20 μm). **(D)** Results of flow cytometry measuring the cellular uptake efficiency in RGCs in each group. **(E)** Scheme of PACAP38 and EXO_*PACAP38*_ delivery after intravitreal injection. **(F)** Representative images of retinal sections showing the delivery efficiency of PACAP38 and EXO_*PACAP38*_. The retinal sections are from animals at 2 and 6 h after injection, and counterstained with RBPMS (green) and DAPI (blue) (scale bar = 20 μm) (NC: normal control).

To load PACAP38 on EXOs, we synthesized a chimeric peptide consisting of PACAP38 and CP05 and incubated it with EXOs for 4 h (Figure 1A). The FACS results showed that PACAP38 was efficiently loaded onto EXOs, with approximately 87.1% binding efficiency (Figure 1B). Consistent with previous reports (Gao et al., 2018), TEM showed a sauce-cup shape, with a size range of 30–150 nm (Supplementary Figures 1A,B). Exosome marker proteins, including Alix, CD63, CD81, and CD9 (Gao et al., 2018), were found to be expressed in EXOs, but not cytochrome C, a marker for mitochondria (Supplementary Figure 1C), indicating that there was no contamination of organelles. Notably, significantly increased fluorescence intensity was found in RGCs incubated with EXO_*PACAP38*_, in which EXOs were labeled with DiR and PACAP38 was labeled with FITC, compared with RGCs treated with the mixture of EXOs and PACAP38 without CP05 (Figure 1C). Consistently, the FACS results showed up to 86.9% uptake in RGCs treated with EXO_*PACAP38*_ (Figure 1D), indicating that EXOs mediate the efficient delivery of PACAP38 to RGCs.

To determine whether EXOs could have a similar impact *in vivo*, we intravitreally injected rhodamine-labeled PACAP38 or EXOs loaded with rhodamine-labeled PACAP38 (rhodamine-EXO_*PACAP38*_) in rats (Figure 1E). The results showed that EXO_*PACAP38*_ was preferentially taken up by RGCs over PACAP38 at 2 and 6 h after injection (Figure 1F). These data indicate that CP05 mediates the efficient loading of PACAP38 on EXOs, and EXOs promote the delivery of PACAP38 to RGCs *in vivo*.

### EXO_*PACAP38*_ Enhances the Survival of RGCs in TON Rats

**FIGURE 2:**
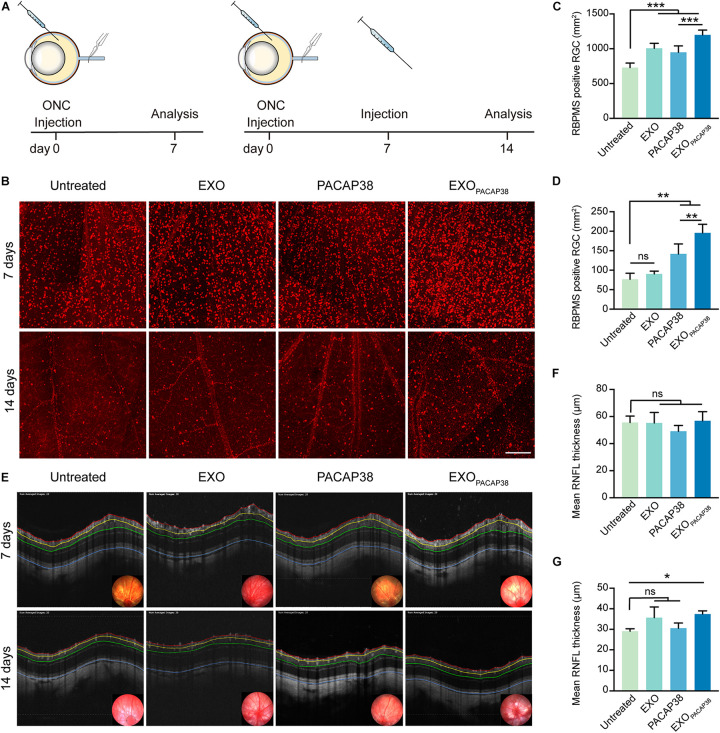
Examination of the survival of RGCs in EXO_*PACAP38*_-treated rat retinas after ONC. **(A)** Experimental timeline. ONC was performed on rats followed by intravitreal injection of EXOs, PACAP38, and EXO_*PACAP38*_. After 7 days, some rats were sacrificed. The remaining rats were given a second injection and sacrificed at 14 days after injury. **(B)** Representative fluorescence images of RBPMS-labeled RGCs in a 1-mm^2^ region of the retina at 7 and 14 days after injury, divided into the untreated, EXO-treated, PACAP38-treated, and EXO_*PACAP38*_-treated groups (scale bar = 200 μm). **(C)** Quantitative analysis of the number of RGCs at 7 days after injury (*n* = 4–5; values are mean ± SEM, one-way ANOVA, ***P < 0.001). **(D)** Quantitative analysis of the number of RGCs at 14 days after injury (*n* = 4; values are mean ± SEM, one-way ANOVA, **P < 0.01). **(E)** Representative OCT images showing the thickness of different layers of the retina within a range of 3,600 μm in circumference with the optic disc as the center (black circle) at 7 and 14 days after injury, divided into the untreated, EXO-treated, PACAP38-treated, and EXO_*PACAP38*_-treated groups. RNFL refers to the distance between the red and yellow lines. **(F)** Quantitative analysis of the mean RNFL thickness (μm) of rats at 7 days after injury (*n* = 3, values are mean ± SEM, one-way ANOVA). **(G)** Quantitative analysis of the mean RNFL thickness (μm) of rats at 14 days after injury (*n* = 3; values are mean ± SEM, one-way ANOVA, *P < 0.05).

To mimic the conditions of TON, we adopted a commonly used ONC injury rat model, which is characterized by axonal degeneration and subsequent loss of neurons dominated by RGCs (Tang et al., 2011; Figure 2A). Consistent with previous studies (Nadal-Nicolas et al., 2009, 2015; Galindo-Romero et al., 2013), a significant loss of RBPMS^+^ RGCs was observed 7 days after injury (729 ± 28.96/mm^2^ of the retina) and 14 days after injury (76.84 ± 7.63/mm^2^ of the retina), compared with that in uninjured rats (2070.35 ± 54.42/mm^2^ of the retina; Supplementary Figure 1D), indicating massive death of RGCs within 2 weeks after ONC in rats. We next investigated whether EXO_*PACAP38*_ could prevent the progressive loss of RGCs in TON rats. We administered EXOs, PACAP, and EXO_*PACAP38*_ into the retinas of TON rats and examined RBPMS^+^ RGCs at 7 or 14 days after injury using the retinal whole-mount technique. Strikingly, significantly more RBPMS^+^ RGCs were observed in EXO_*PACAP38*_-treated rats, at about 1198.90 ± 28.06/mm^2^ of the retina, whereas 729.00 ± 28.96, 1007.03 ± 28.96, and 949.69 ± 37.40/mm^2^ were found in the untreated group, EXO-treated group, and PACAP38-treated group of TON rats at 7 days after injury (Figures 2B,C). Corroborating the data of day 7, significantly more RBPMS^+^ RGCs were found in the EXO_*PACAP38*_-treated TON rats (195.63 ± 11.09/mm^2^) than in the untreated, EXO-treated, and PACAP38-treated rats (76.84 ± 7.63, 90.48 ± 3.60, and 141.79 ± 12.77/mm^2^, respectively) at 14 days after injury (Figures 2B,D). These data demonstrate that EXO_*PACAP38*_ significantly promotes RGC survival at 7 and 14 days after injury.

### EXO_*PACAP38*_ Preserves the RNFL Thickness in TON Rats

The thickness of the RNFL is an important parameter for the axonal density of RGCs (Mead and Tomarev, 2017). To evaluate whether EXO_*PACAP38*_ can preserve the thickness of the RNFL, we examined RNFL thickness using OCT, a noninvasive method for assessing degenerative changes in the converging axons of RGCs. The results showed a much less reduction in RNFL thickness in TON rats treated with EXO_*PACAP38*_, with a thickness of 37.44 ± 0.90 μm, than in rats treated with EXOs (35.70 ± 2.99 μm), rats treated with PACAP38 (30.50 ± 1.48 μm), and untreated control rats (29.09 ± 0.69 μm) at 14 days after injury (Figures 2E,F). In contrast, the change in the RNFL thickness among the groups at 7 days after injury was minor (Figures 2E,G), suggesting that 14 days after injury is a better time point for subsequent experiments, which is consistent with previous reports (Mead and Tomarev, 2017). Altogether, these results demonstrate that EXO_*PACAP38*_ can delay RNFL loss.

### EXO_*PACAP38*_ Promotes RGC Axon Regeneration in TON Rats

**FIGURE 3:**
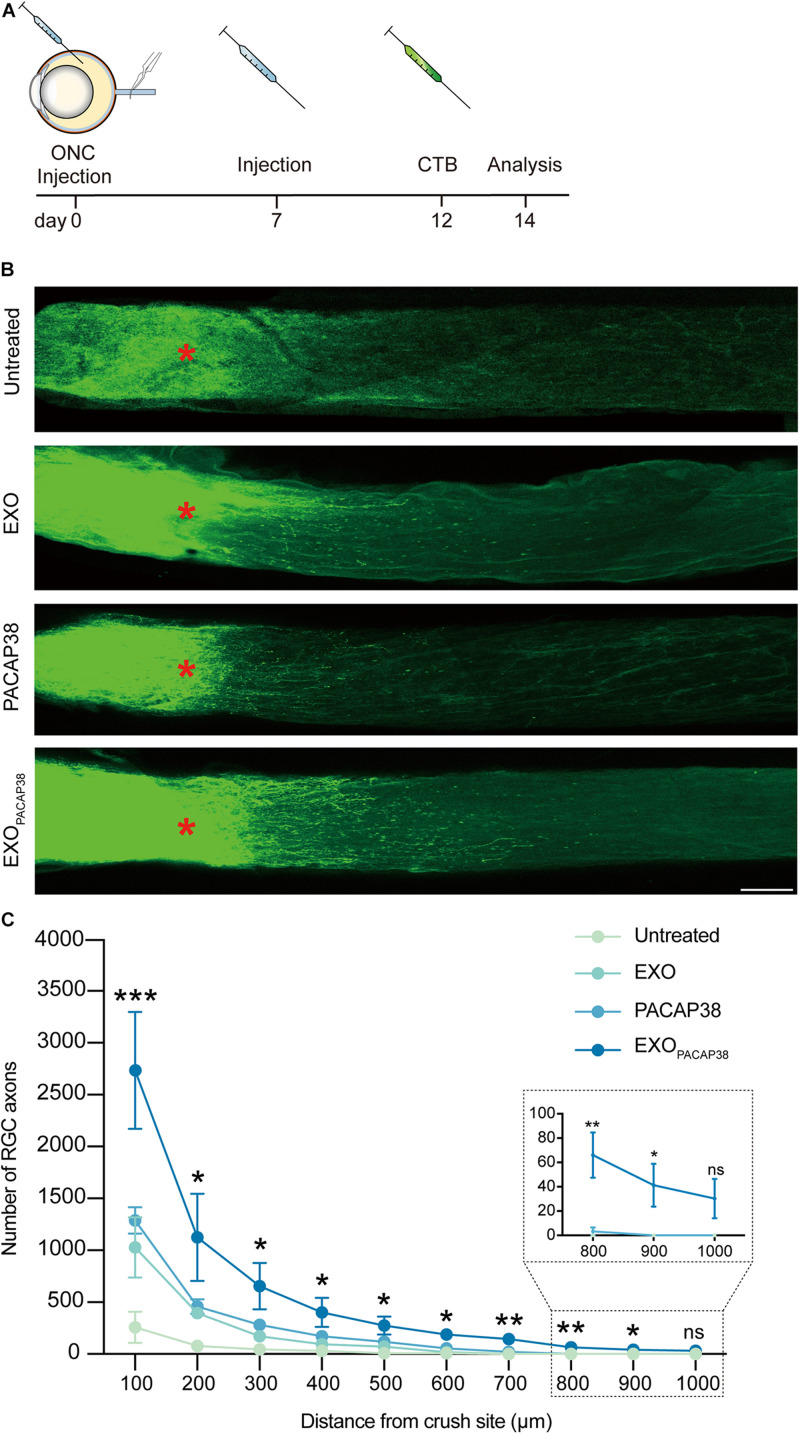
Evaluation of optic nerve regeneration in EXO_*PACAP38*_-treated rats after ONC. **(A)** Experimental timeline. ONC was performed on rats followed by intravitreal injection of EXOs, PACAP38, and EXO_*PACAP38*_. After 7 days, the rats were given a second injection. Axons were traced with CTB-conjugated Alexa Fluor 488 at 2 days before animal sacrifice. **(B)** Laser scanning confocal microscope images showing the regeneration of optic nerve axons at 14 days after injury, divided into the untreated, EXO-treated, PACAP38-treated, and EXO_*PACAP38*_-treated groups (scale bar = 100 μm). Optic nerve axons were traced using CTB. Asterisks indicate the ONC site. **(C)** Quantitative analysis of optic nerve axon number in the range of 100–1,000 μm from the crush site (red asterisks in B) (*n* = 4; one-way ANOVA on Tukey’s multiple comparisons test,*P < 0.05, **P < 0.01, ***P < 0.001, relative to the untreated group).

Cholera toxin subunit B (CTB) can be used to trace neurites including RGC axons in an antegrade or retrograde manner, and can be used as a marker for evaluating the damage and regeneration of RGC axons (Lanciego and Wouterlood, 2011; de Sousa et al., 2013; Duan et al., 2015). We examined axon regeneration with CTB-conjugated Alexa Fluor 488 in TON rats 14 days after injury (Figure 3A). Strikingly, much stronger fluorescence signals of CTB far from the crush site were detected in EXO_*PACAP38*_-treated TON rats than in EXO- or PACAP38-treated rats. In contrast, there was no fluorescence signal of CTB far from the injury site in the untreated group (Figure 3B). A distance of 0.5 mm from the crush site was established as the optimal area for examining axon regeneration (Mak et al., 2020). A significant increase in the number of RGC axons (275.75 ± 88.12) was detected in the nerves (at least with a distance of 0.5 mm to the crush site) of TON rats treated with EXO_*PACAP38*_ compared with rats treated with PACAP38 (117.00 ± 43.91), rats treated with EXOs (71.00 ± 24.05), or untreated control rats (9.50 ± 9.50) under identical conditions. Although we observed differences, only the EXO_*PACAP38*_ group showed a statistically significantly enhanced axon regeneration compared with the untreated group (Figure 3C). These data demonstrate that EXO_*PACAP38*_ significantly promotes axon regeneration at 14 days after injury.

### EXO_*PACAP38*_ Improves Optic Nerve Function in TON Rats

**FIGURE 4:**
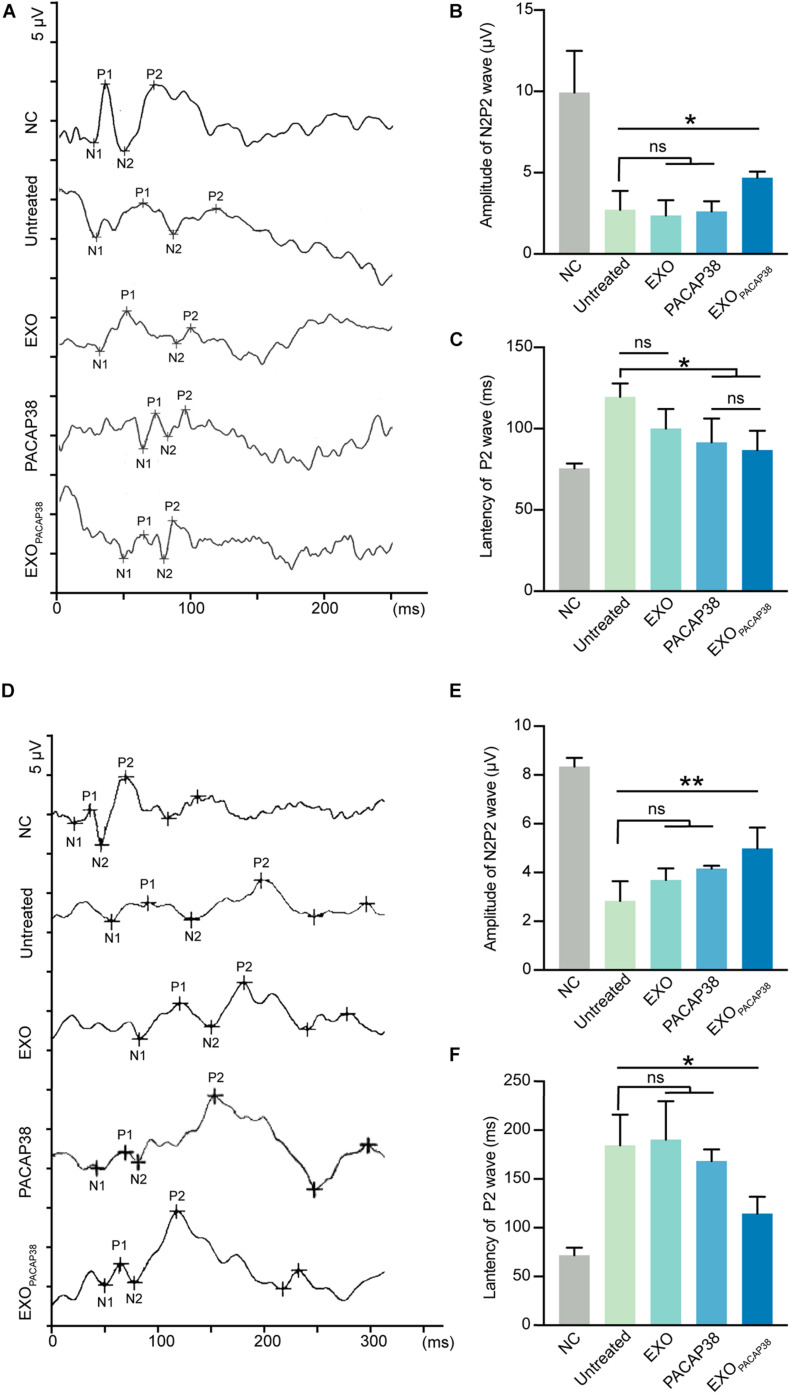
Evaluation of visual function in EXO_*PACAP38*_-treated rats after ONC. **(A)** F-VEP was recorded at 7 days after injury in normal controls; untreated group; and EXO-, PACAP38-, and EXO_*PACAP38*_-treated groups. **(B)** Analysis of N2P2 wave at 7 days after injury (*n* = 4; values are mean ± SEM, one-way ANOVA, *P < 0.05). **(C)** Analysis of P2-wave latencies at 7 days after injury. No statistical significance in P2-wave amplitudes was observed at 7 days after injury in the EXO_*PACAP38*_-treated group compared with the untreated group (*n* = 4; values are mean ± SEM, one-way ANOVA, *P < 0.05). **(D)** F-VEP was recorded at 14 days after injury in normal controls; untreated group; and EXO-, PACAP38-, and EXO_*PACAP38*_-treated groups. **(E)** Analysis of N2P2 wave at 14 days after injury (*n* = 4; values are mean ± SEM, one-way ANOVA, **P < 0.01). **(F)** Analysis of P2-wave latencies at 14 days after injury. Significant improvement in P2-wave latencies was achieved at 14 days after injury in the EXO_*PACAP38*_-treated group compared with the untreated group (*n* = 4; values are mean ± SEM, one-way ANOVA, *P < 0.05).

Changes in the functional properties of RGCs after ONC were tested by measuring the F-VEP. The amplitude and latency of the P2-wave are measures of optic nerve function (Chien et al., 2016). As expected, intravitreal injection of PACAP38 (2.62 ± 0.31 μV) or EXO_*PACAP38*_ (4.7 ± 0.19 μV) improved the amplitude of the P2-wave at 7 days after injury compared with no treatment (2.72 ± 0.58 μV), whereas EXO treatment (2.37 ± 0.54 μV) caused no difference. Furthermore, at 7 days after injury, the latency of the P2-wave was significantly decreased in TON rats. The latency of the P2-wave increased from 75.5 ± 1.55 to 119.5 ± 4.17 ms at 7 days after injury, whereas EXO_*PACAP38*_ treatment preserved the P2 latency (86.75 ± 5.98 ms). Moreover, the EXO-treated group (100 ± 7 ms) and PACAP38-treated group (91.5 ± 7.35 ms) showed no significant difference from the untreated group (Figures 4A–C).

The results at 14 days after injury showed considerable similarities to those at 7 days. Intravitreal injection of EXO_*PACAP38*_ significantly improved the amplitude of the P2-wave (4.99 ± 0.43 μV) at 14 days after injury, compared with no treatment (2.84 ± 0.41 μV), EXO treatment (3.69 ± 0.24 μV), and PACAP38 treatment (4.16 ± 0.06 μV). Notably, EXO_*PACAP38*_ treatment significantly reduced the P2-wave latency (114.5 ± 8.68 ms) at 14 days after injury compared with PACAP38 treatment (168.5 ± 5.95 ms), EXO treatment (190.25 ± 19.75 ms), and no treatment (184.5 ± 15.76 ms) under identical conditions (Figures 4D–F). These results confirmed the potent effect of EXO_*PACAP38*_ in improving optic nerve function in TON rats.

### EXO_*PACAP38*_ Does Not Elicit Any Detectable Adverse Effect in TON Rats

**FIGURE 5:**
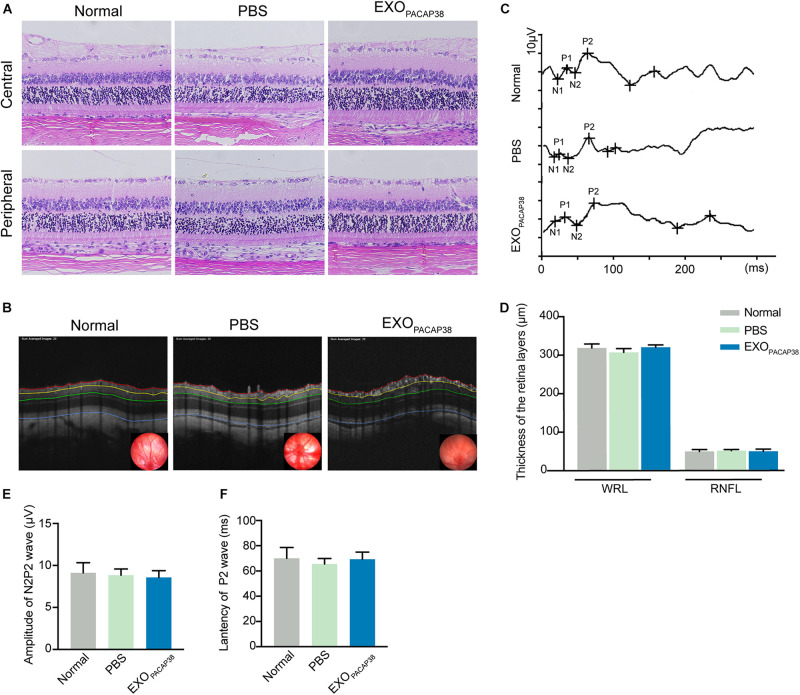
Intravitreal injection of EXO_*PACAP38*_ to rat eyes did not cause any toxicity to the retina. **(A)** H&E staining of retinal sections showing the morphology of the central (top) and peripheral (bottom) parts of retinal tissues in the normal control, vehicle (PBS)-treated, and EXO_*PACAP38*_-treated groups. **(B)** Representative OCT images showing the thickness of different layers of the retina within a range of 3600 μm in circumference with the optic disc as the center (black circle) at 14 days after injury in the normal control, vehicle (PBS)-treated, and EXO_*PACAP38*_-treated groups. RNFL refers to the distance between the red and yellow lines. The whole retinal layer (WRL) refers to the distance between red and blue lines. **(C)** Quantitative analysis of the mean thickness of the RNFL and WRL of rats at 14 days. No significance in the mean thickness of the RNFL or WRL was observed at 14 days among the normal control, PBS-treated, and EXO_*PACAP38*_-treated groups (*n* = 3; values are mean ± SEM, one-way ANOVA). **(D)** F-VEP was recorded at 14 days in the normal control, PBS-treated, and EXO_*PACAP38*_-treated groups. **(E,F)** EXO_*PACAP38*_ did not induce F-VEP changes. No significant improvement in the amplitudes and latencies of the P2-wave was achieved at 14 days after injury among the normal control, PBS-treated, and EXO_*PACAP38*_-treated groups (*n* = 4; values are mean ± SEM, one-way ANOVA).

To investigate whether the administration of EXO_*PACAP38*_ causes any toxicity to the retina and optic nerve, we examined the morphological and structural changes of the retina and optic nerve 14 days after injection. Hematoxylin and eosin (H&E) staining revealed the typical morphology of the cell layers in rats treated with EXO_*PACAP38*_, rats treated with PBS, and negative controls (Figure 5A). OCT was used to examine the changes in retinal thickness in response to a stimulus, and the results showed no difference in the RNFL and whole retinal layer (Figures 5B,D). In addition, there was no change in the F-VEP response of the P2-wave amplitudes among the groups (Figures 5C,E,F). These results indicate that there were no drug-related adverse effects.

## Discussion

In this study, we demonstrated that EXOs could load PACAP38 via CP05 and efficiently transport this functional neuropeptide to the retina in a TON rat model. EXO_*PACAP38*_ improved RGC survival and axon regeneration in response to injury. Importantly, EXOs improved the transport efficiency of the neuroprotective peptide by overcoming its shortcomings of low tissue penetration and short half-life. This study on ophthalmic peptide drug transport showed the capability of exosomes as carriers for efficiently supplementing the endogenous neuroprotective peptide PACAP38 for the treatment of TON. This study also further suggests the theoretical feasibility of using exosomes as a tool for the delivery of different types of neuropeptides that can effectively offset the complex pathological conditions of TON, as a combination therapy.

Notably, some studies have indicated the therapeutic potential of exosomes in several retinal disease models (Mead and Tomarev, 2020). For instance, exosomes released from retinal astrocyte cells contain antiangiogenic proteins such as endostatin and pigment epithelium-derived factor, which may contribute to the inhibition of laser-induced choroidal neovascularization in mice (Hajrasouliha et al., 2013). It is also known that exosomes derived from mesenchymal stem cells are beneficial for RGCs after optic nerve injury. Both exosomes derived from bone marrow mesenchymal stem cells and exosomes derived from umbilical cord mesenchymal stem cells could promote RGC survival depending on their miRNA cargo (Mead and Tomarev, 2017; Pan et al., 2019). However, neither of the mesenchymal stem cell-derived exosomes specifically targets RGCs. In our study, we aimed to efficiently deliver the neuroprotective peptide to RGCs. Several studies have demonstrated that autologous exosomes could be used as ideal vehicles for the delivery of therapeutic agents to parent cells, with a homing effect. It has been shown that drug-loaded glioma cell (GM)-derived exosomes inhibited the proliferation of parent GMs more than they inhibited heterologous GMs (Thakur et al., 2020). It has also been shown that cell-type tropism leads to “homing” of cancer cell-derived exosomes and their preferential uptake by the donor cancer cells and tumor-associated immune cells in solid tumors (Emam et al., 2019). At the same time, recent studies have shown that replacing lost or damaged RGCs with healthy RGCs or RGC precursors could facilitate the development and enhancement of connections to ganglion cells and optic nerve axons (Behtaj et al., 2020), and that healthy neuronal exosomes can regulate the development of neural circuits and have great potential to repair damaged brain cells (Sharma et al., 2019). Accordingly, we chose RGC-derived exosomes as delivery carriers in our study.

The neuroprotective and axogenic effects of PACAP have been proven in numerous animal models of neurological diseases, such as cerebral ischemia, Alzheimer’s disease, and traumatic brain injury (Han et al., 2014; Matsumoto et al., 2016; Toth et al., 2020). In the retina, PACAP has been shown to attenuate RGC apoptosis. However, multiple intravitreal injections are required within a short time period (Ye et al., 2019). Therefore, loading the peptide to EXOs provides a better solution.

In our study, we observed that EXO_*PACAP38*_ had a high affinity for RGCs *in vitro* and *in vivo.* In the TON model, EXO_*PACAP38*_ significantly enhanced RGC survival and preserved RNFL thickness. It is interesting that EXOs slightly rescued the loss of RGCs at first. We hypothesized that healthy neuronal exosomes might have the potential to rescue damaged neuronal cells, as previously discussed. In addition, RNFL thickness measurements showed no significant difference among groups until 14 days after injury, which might be explained by the acute responses to ONC injury including tissue edema, as reported in previous studies (Nagata et al., 2009; Li et al., 2020).

Axonal regeneration is essential to restore neuronal connectivity and to reestablish the function of the visual system. As in other parts of the central nervous system (CNS) in adult mammals, injured axons in the optic nerve do not spontaneously regenerate after injury (Williams et al., 2020). Previous studies have identified several crucial regulators of the intrinsic regenerative ability of RGCs using mouse genetics, transcriptomics, and viral vectors (Williams et al., 2020). For instance, genetic deletion of *KLF4* in RGCs increased the number of regenerating axons at multiple distances from the injury site (Moore et al., 2009). Another study showed that manipulating the ectopic expression of *Oct4* through the adeno-associated virus (AAV) system in mouse RGCs restores youthful DNA methylation patterns and transcriptomes and promotes axon regeneration after injury (Lu et al., 2020). Although the gene therapy strategy have achieved positive results in some clinical and preclinical settings, there are still potential risks such as immune responses and genomic changes (Mingozzi and High, 2013; Nguyen et al., 2021). Exosomes are natural products of the body that induce a low immune response and has a high safety profile. In the current study, we evaluated the axon regeneration effects of EXOs, PACAP38, and EXO_*PACAP38*_. The results showed that only EXO_*PACAP38*_ promoted robust regeneration of axons and preserved the function of RGCs. PACAP has been demonstrated to promote peripheral nerve repair after injury (Woodley et al., 2019; Baskozos et al., 2020); however, it is dysfunctional in the CNS (Ye et al., 2019). These results indicate that this system may strengthen the biological function of the peptide. In a previous study, we successfully loaded two peptides with distinctive functions onto exosomes (Gao et al., 2018). Thus, further studies may be conducted to test whether better therapeutic outcomes can be achieved by loading more types of peptides with different biological functions using this delivery system.

In conclusion, we demonstrated that exosomes derived from rat RGCs can be employed as a new biological nano-drug-loading system for the neuroprotective peptide PACAP38. The connection complex, EXO_*PACAP38*,_ can mediate high-efficiency neuroprotection and has axon regeneration effects, providing an approach for future clinical translation.

## Data Availability Statement

The data that support the findings of this study is available from the corresponding author upon reasonable request.

## Ethics Statement

The animal study was reviewed and approved by the Tianjin Medical University Animal Care and Use Committee.

## Author Contributions

TW, HFY, and HY designed the project. TW, YL, MG, XD, and ML performed the experiments. TW and YL analyzed the data and wrote the manuscript. MD, XW, HFY, and HY reviewed and edited the manuscript. All authors discussed the results and approved the submitted version.

## Conflict of Interest

The authors declare that the research was conducted in the absence of any commercial or financial relationships that could be construed as a potential conflict of interest.

## Supplementary Material

Supplementary Figure 1 Characterization of exosomes derived from rat RGCs. (A) Transmission electron microscopy image of the EXOs (scale bar = 100 nm). (B) Nanoparticle tracking analysis detection of the size distribution of EXOs. (C) Western blot detection showing the expression of exosomal biomarkers in EXOs. The total protein (20 or 40 μg) from RGC lysates or EXOs was loaded, and cytochrome C was used as an organelle marker. Sup: supernatant; RGC: referring to the RGC lysate. (D) Whole mount of an intact retina of 1 mm^2^ (scale bar = 200 μm). (E) Quantitative analysis of the number of RGCs at 7 and 14 days after injury (dpi: days post injury; *n* = 3, values are mean ± SEM, one-way ANOVA, ^∗∗∗^*P* < 0.01).
