# Supplementary figures and images for: Exosome-Mediated Delivery of the Neuroprotective Peptide PACAP38 Promotes Retinal Ganglion Cell Survival and Axon Regeneration in Rats With Traumatic Optic Neuropathy
Source: Front Cell Dev Biol. 2021 Apr 6;9:659783. doi: 10.3389/fcell.2021.659783 (PMC8055942; doi:10.3389/fcell.2021.659783)

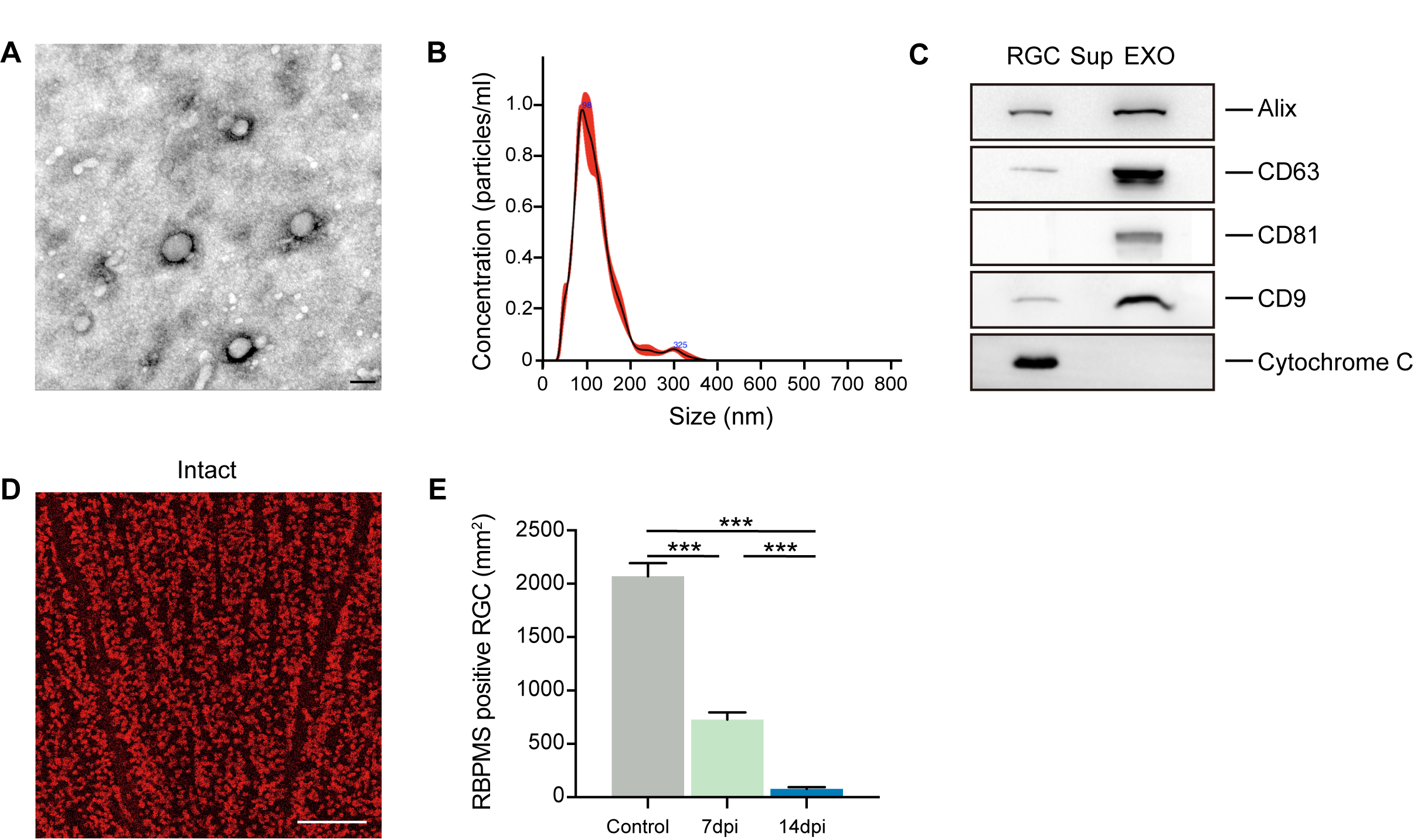

Supplement: Supplementary Figure 1 — Characterization of exosomes derived from rat RGCs. (A) Transmission electron microscopy image of the EXOs (scale bar = 100 nm). (B) Nanoparticle tracking analysis detection of the size distribution of EXOs. (C) Western blot detection showing the expression of exosomal biomarkers in EXOs. The total protein (20 or 40 μg) from RGC lysates or EXOs was loaded, and cytochrome C was used as an organelle marker. Sup: supernatant; RGC: referring to the RGC lysate. (D) Whole mount of an intact retina of 1 mm2 (scale bar = 200 μm). (E) Quantitative analysis of the number of RGCs at 7 and 14 days after injury (dpi: days post injury; n = 3, values are mean ± SEM, one-way ANOVA, ∗∗∗P < 0.01). [file Image_1.TIF]
